# Supplementary material for: Targeting Key Signaling Pathways in Glioblastoma Stem Cells for the Development of Efficient Chemo- and Immunotherapy
Source: Int J Mol Sci. 2022 Oct 26;23(21):12919. doi: 10.3390/ijms232112919 (PMC9659205; doi:10.3390/ijms232112919)
Supplement: Supplementary file 1 [file ijms-23-12919-s001.zip › ijms-1924193-supplementary.pdf]

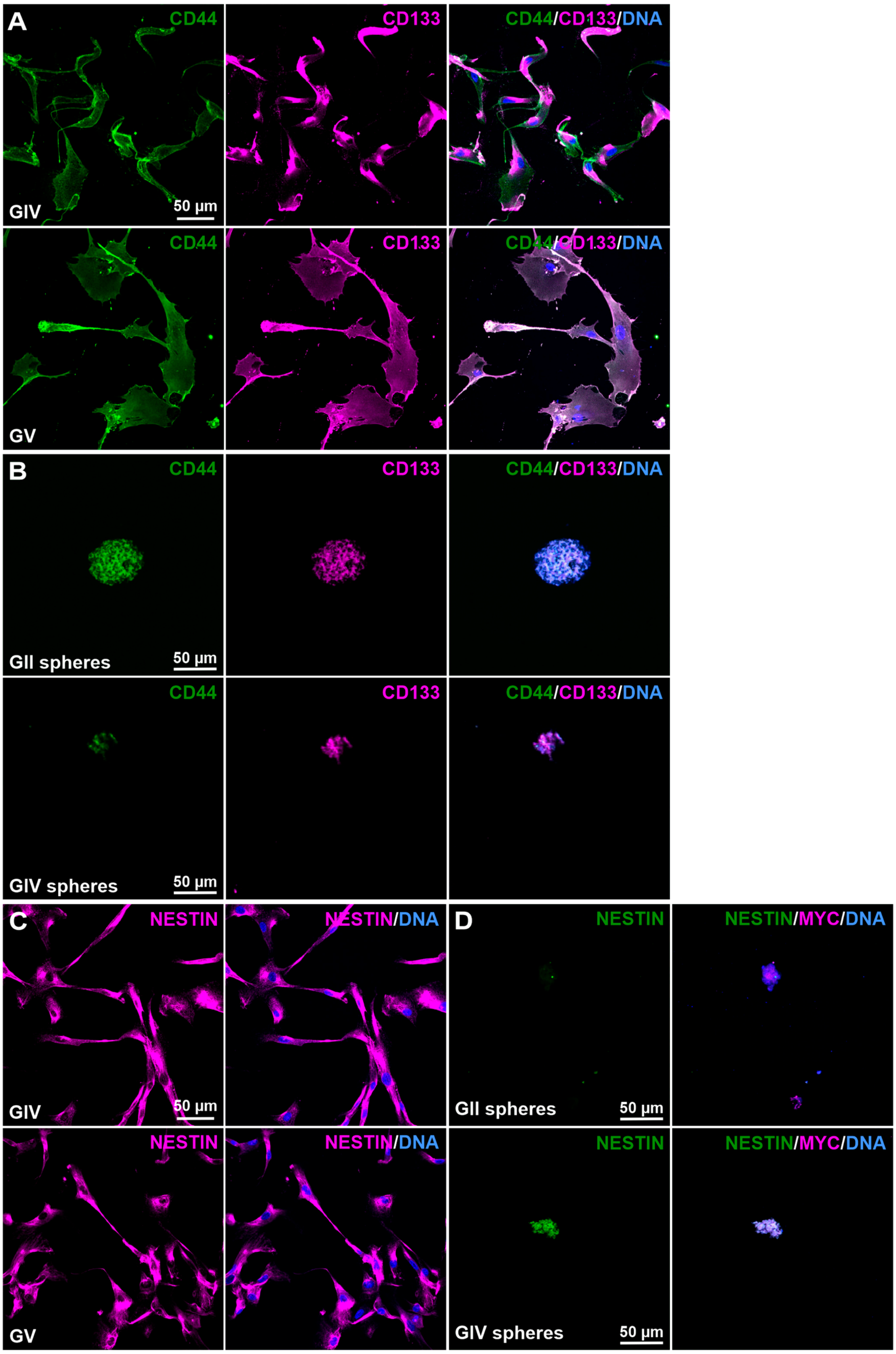

Figure S1: Characterization of GSCs.

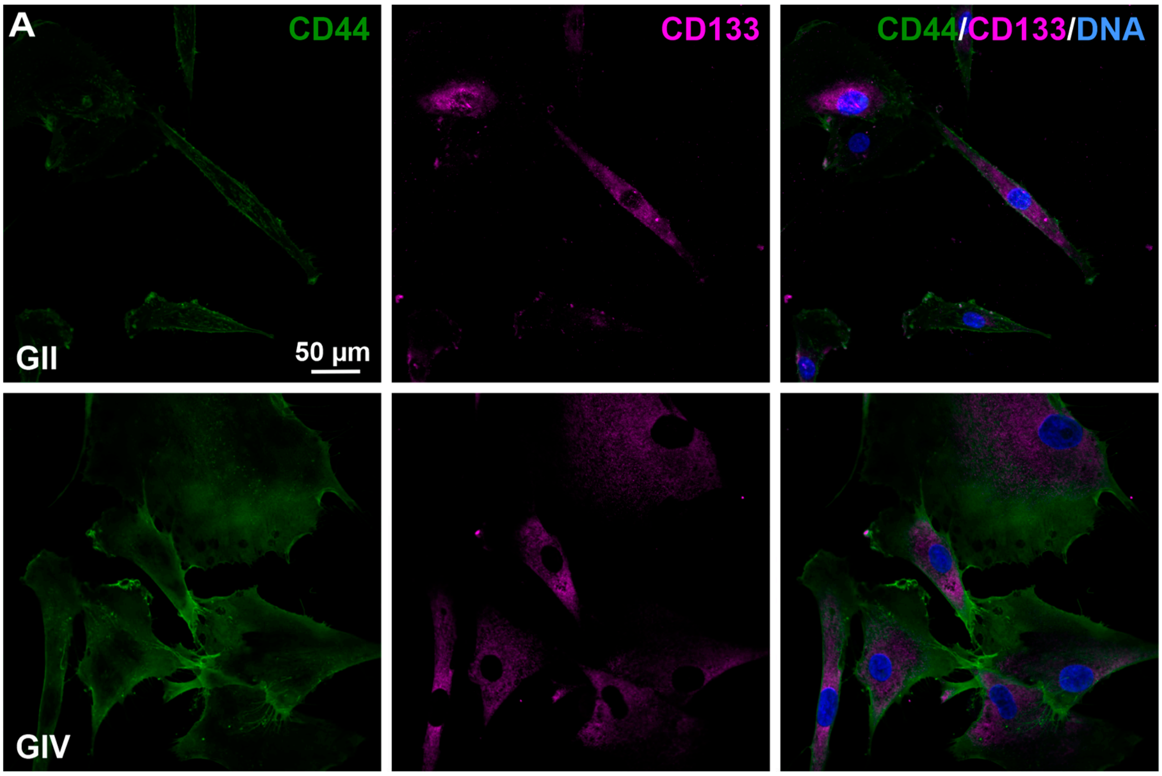

Figure S2: High passage GSCs express CD133, CD4 and Nestin.

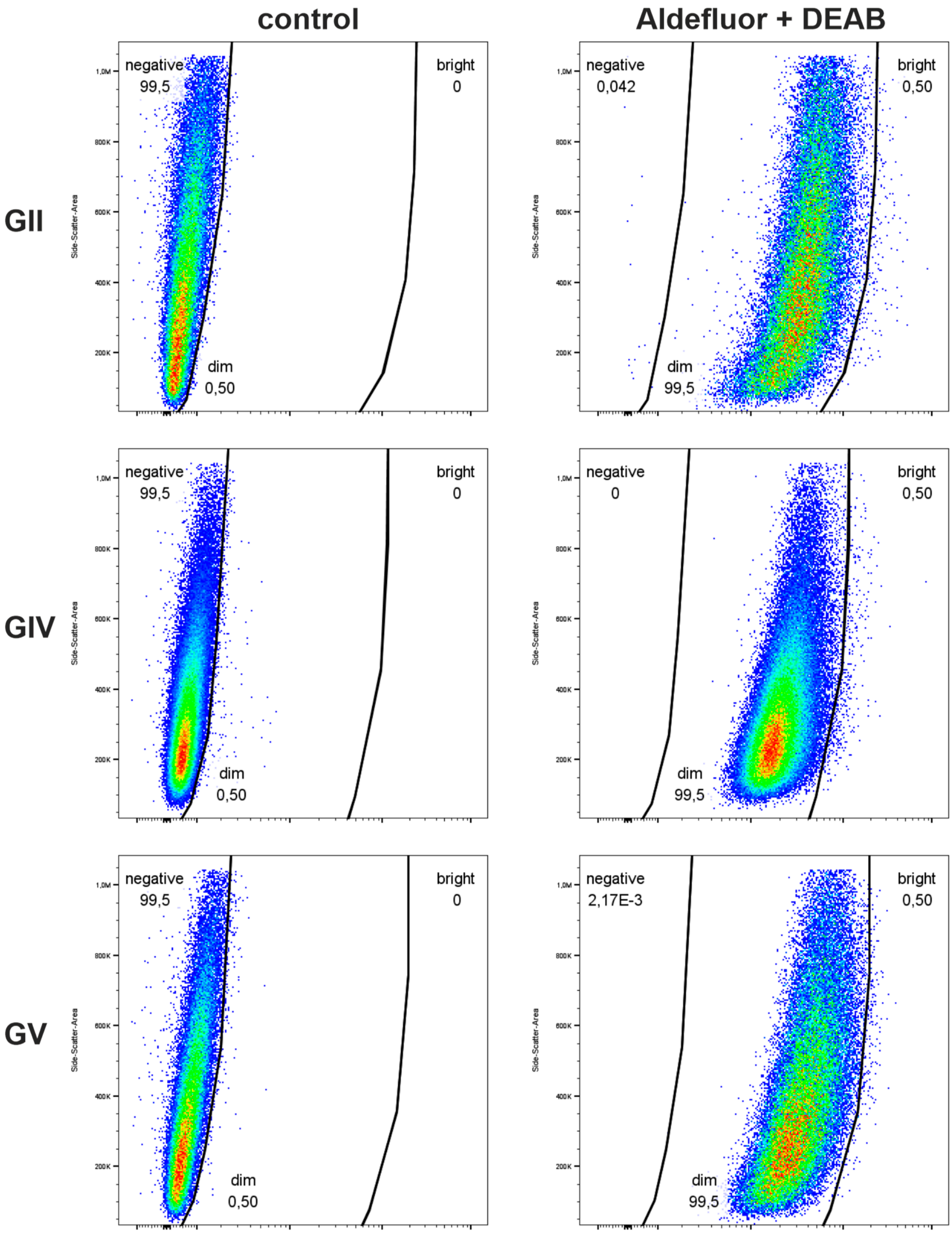

Figure S3: DEAB controls of flow cytometric-analyzed ALDH1 activity.

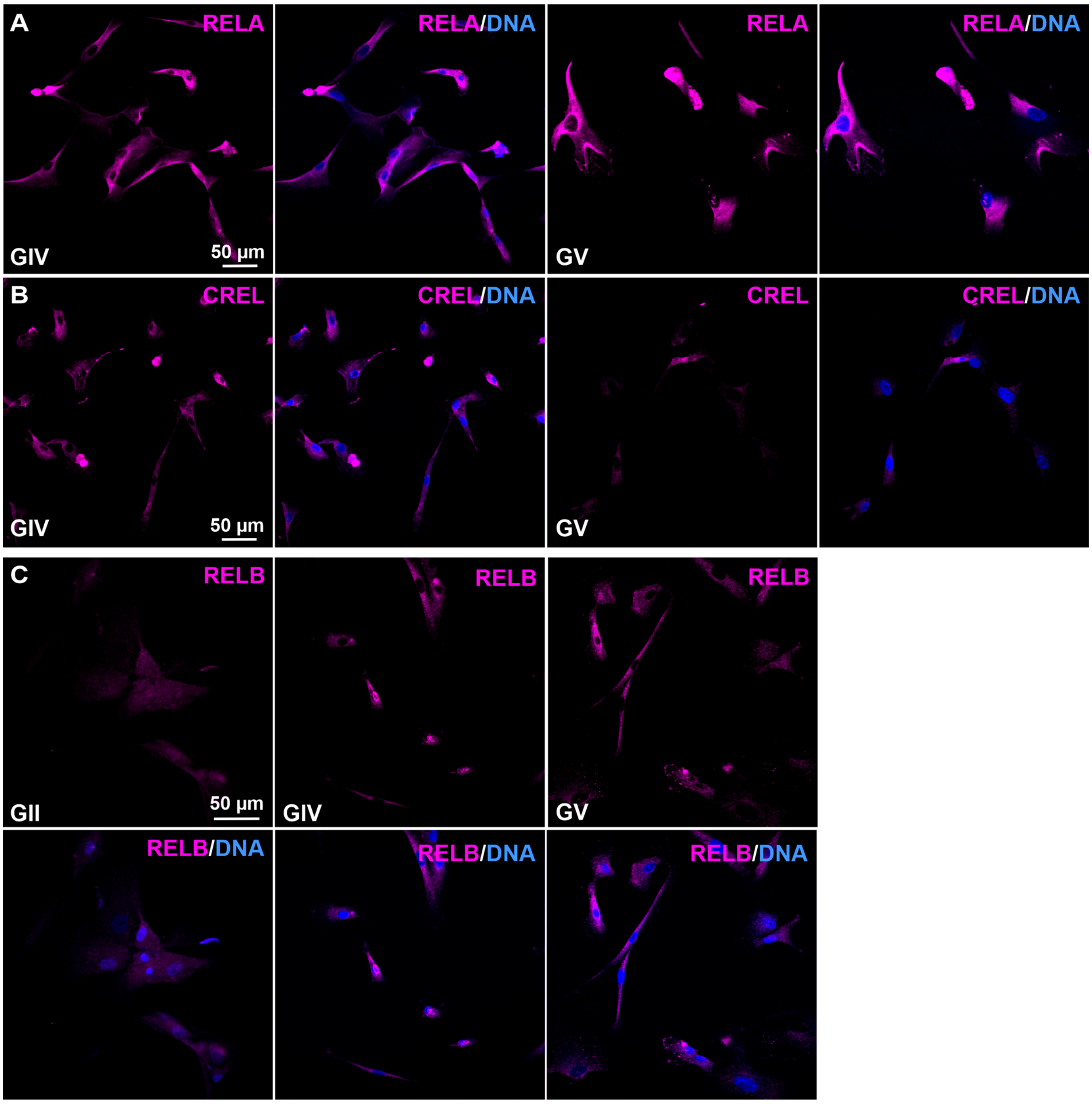

Figure S4: GSCs express NF-κB subunits.

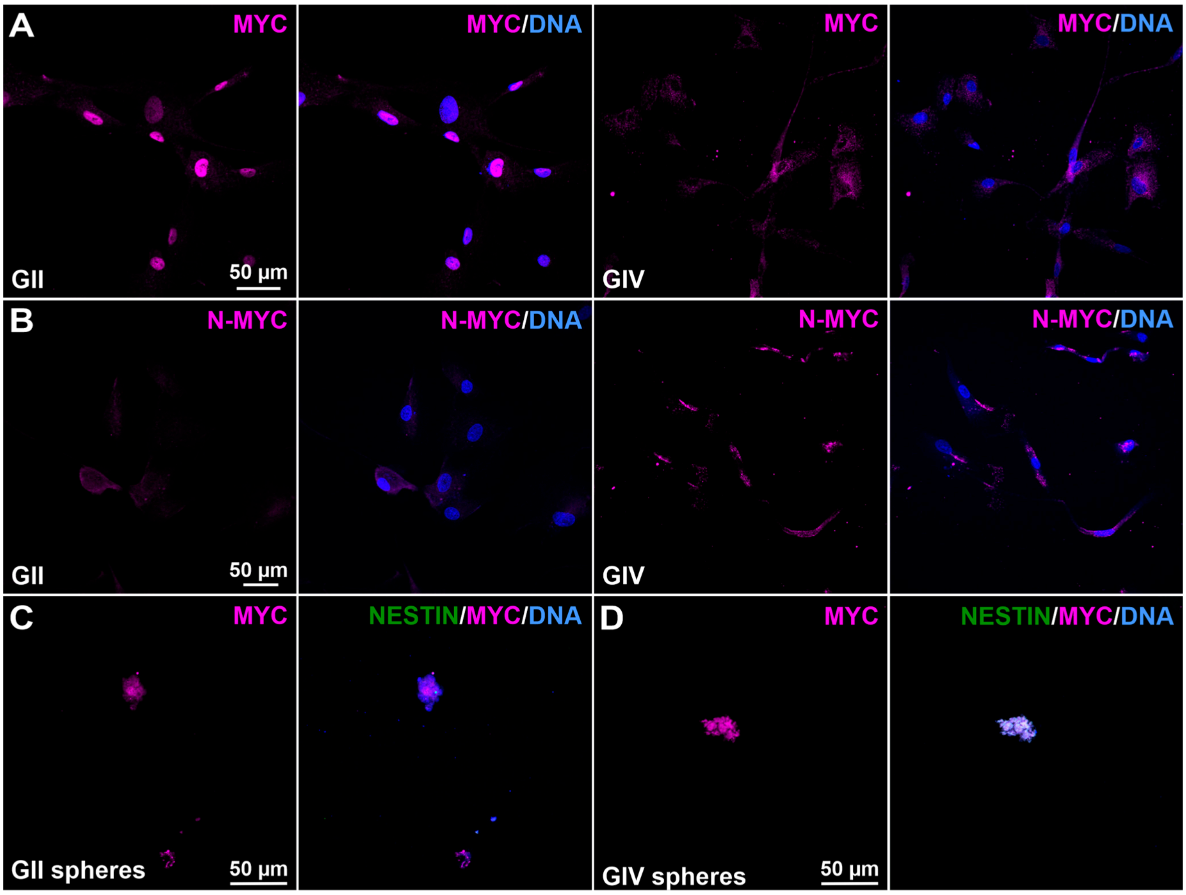

Figure S5: GSCs express MYC and N-MYC.

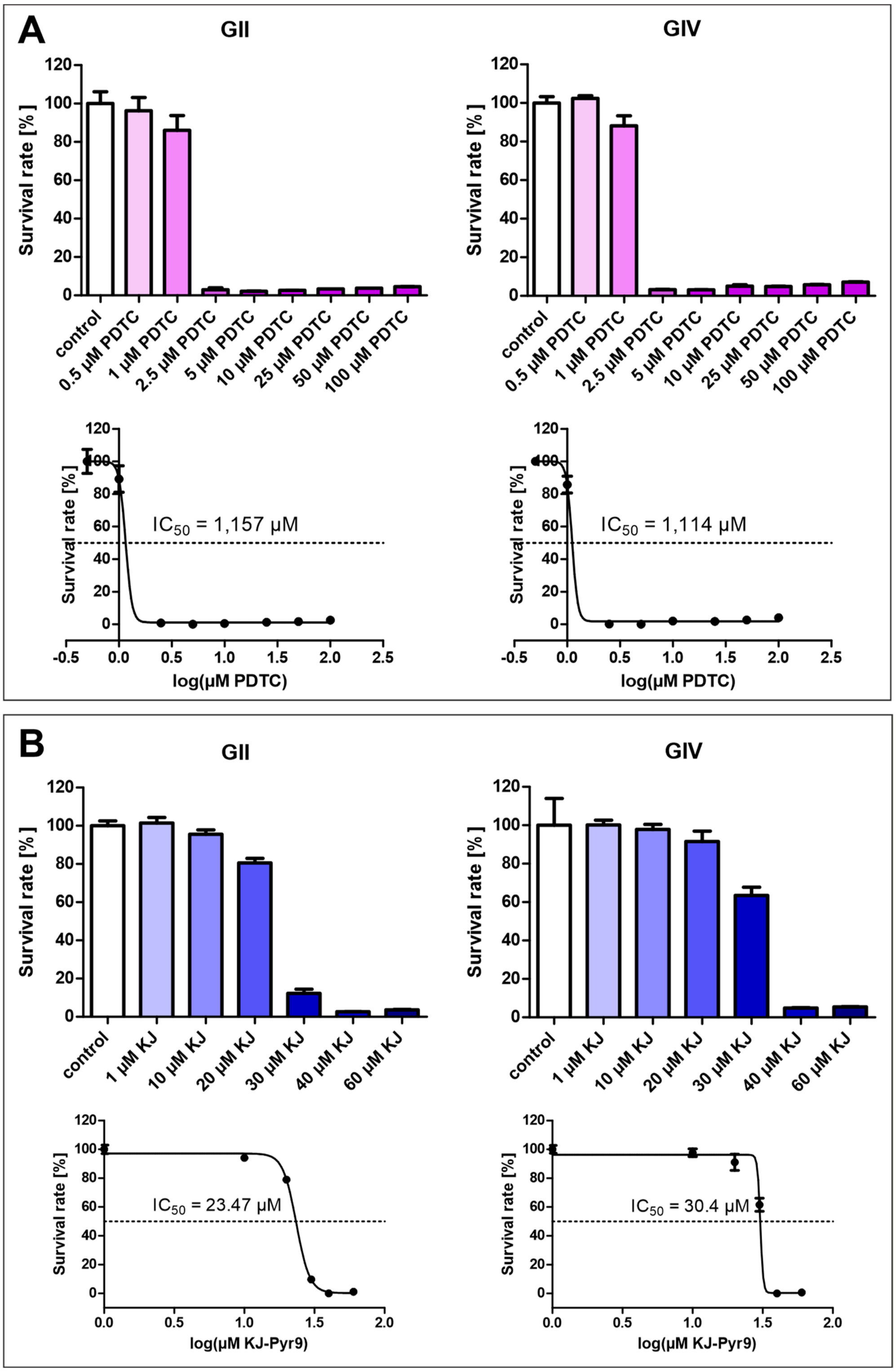

Figure S6: PDTC and KJ-Pyr-9 treatment with the respective IC_{50} plot.

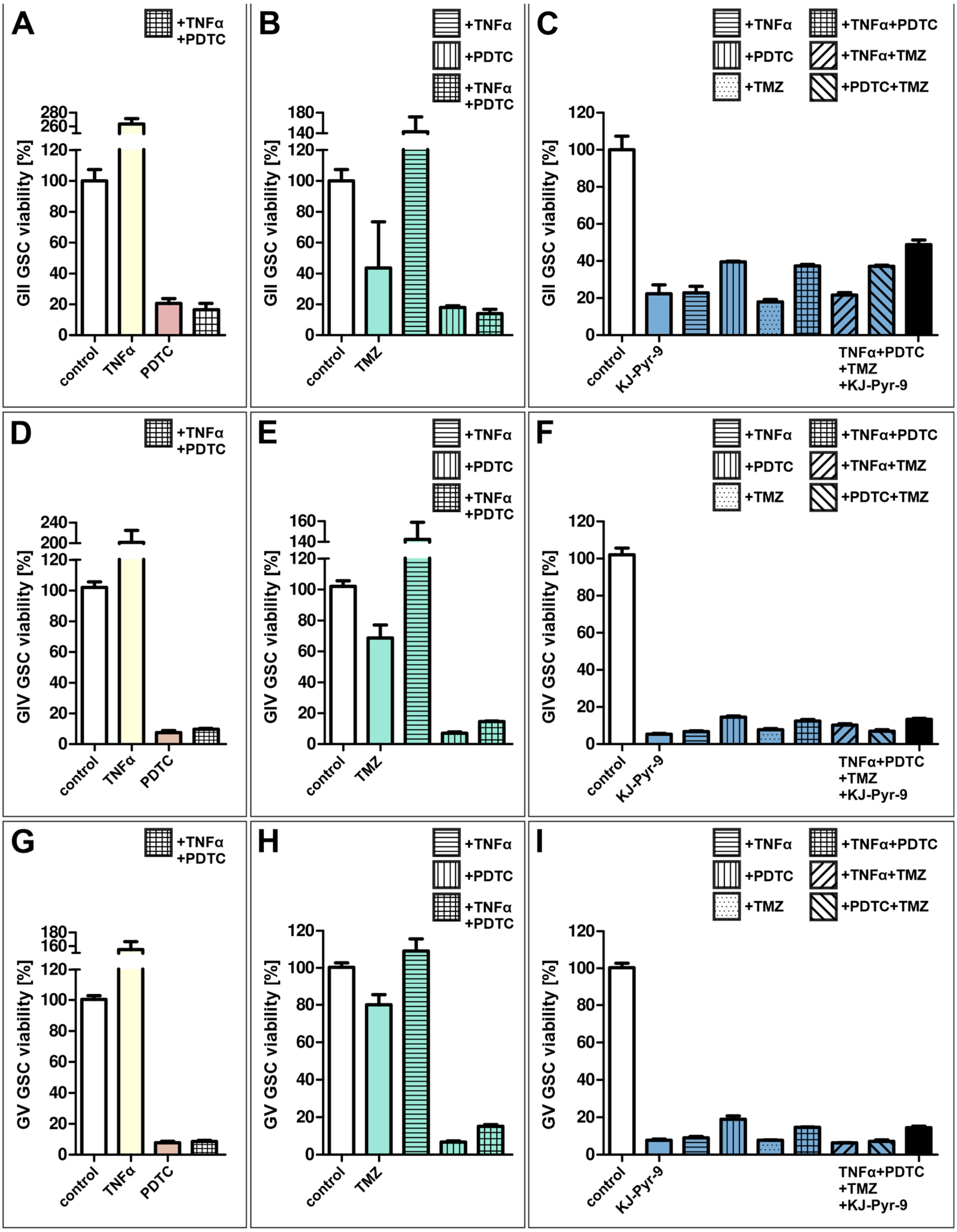

Figure S7: Inhibition of NF-κB and MYC impairs GSC viability.
